# Molecular Investigation of the Antitumor Effects of Monoamine Oxidase Inhibitors in Breast Cancer Cells

**DOI:** 10.1155/2023/2592691

**Published:** 2023-10-05

**Authors:** Aseel Alkhawaldeh, Sanaa Bardaweel

**Affiliations:** Department of Pharmaceutical Sciences, School of Pharmacy, University of Jordan, Queen Rania Street, Amman 11942, Jordan

## Abstract

The catalytic activity of monoamine oxidase A (MAO-A) has been linked to tumorigenesis due to the production of reactive oxygen species (ROS) and the resulting oxidative stress. MAO-A inhibition revealed a beneficial role in prostate and lung cancer treatment. This study is aimed at evaluating the effect of different monoamine oxidase A inhibitors (MAO-AIs) on the proliferation and progression of breast cancer cell lines. The cell viability assay was used to evaluate the antiproliferative and combined effects of MAO-AIs. Cell migration was evaluated using wound healing, invasion, and colony formation assays. The underlying mechanism of cell death was studied using flow cytometry. The real-time polymerase chain reaction was used to determine the relative gene expression. Finally, MAO-A activity in breast cancer cells was evaluated using an MAO-A activity assay. According to the results, the examined MAO-AIs significantly inhibited the proliferation of breast cancer cells in a dose-dependent manner. In breast cancer cells, the combination of anticancer drugs (doxorubicin or raloxifene) with MAO-AIs resulted in a synergistic effect. MAO-AIs significantly reduced wound closure and invasion ability in breast cancer cells. Also, MAO-AIs reduced the colony count and size of breast cancer cells. MAO-AIs resulted in significant proapoptotic activity in breast cancer cells. Finally, the MAO-AIs suppressed *MAO-A*, *Bcl-2*, and *VEGF* gene expressions in breast cancer cells relative to untreated cells. This study provides solid evidence supporting the anticancer effect of MAO-A inhibitors in breast cancer cells.

## 1. Introduction

The first monoamine oxidase (MAO) enzyme was discovered in 1928 and was called tyramine oxidase. MAO enzyme is a mitochondrial-bound flavin protein that is involved in oxidative deamination reactions. Monoamine oxidase A (MAO-A) and monoamine oxidase B (MAO-B) are key isoenzymes that degrade biogenic and dietary amines [1]. MAO-A is involved in tumorigenesis, cardiovascular diseases, diabetes, and obesity in addition to its importance in brain function [2–5]. MAO-A enzyme causes deoxyribonucleic acid (DNA) damage [4, 6] and oxidative cell injury [6]. Additionally, it may be involved in tumorigenesis via reactive oxygen species (ROS) production from oxidative deamination reactions [7]. The abnormally elevated levels of MAO-A activity result in increased production of harmful byproducts: hydrogen peroxide (H_2_O_2_) and aldehydes [8]. The excessive byproducts lead to cellular oxidative stress that causes mitochondrial toxicity, severe lipid membrane damage, and DNA damage [4], which may lead to cancer development [2, 7]. Interestingly, oxidative stress is related to depression and cancer [7, 9] and is known to be a successful therapy against psychiatric depression using MAO-A inhibitors. Evidence has been growing to support the correlation between MAO-A isoenzyme and many types of cancer, including prostate cancer [2, 10–13], hepatocellular carcinoma [14, 15], brain tumor (gliomas) [16], classical Hodgkin's lymphoma [17], colorectal cancer [18], lung cancer [19–21], and breast cancer [22]. MAO-A inhibition resulted in suppression of the epithelial-to-mesenchymal transformation in A549 and H1299 non-small-cell lung carcinoma cells [23] and demonstrated significant effects in prostate cancer [24, 25]. On the other hand, MAO-A inhibition initiated a mesenchymal-to-epithelial transformation in the breast cancer cell line (MDA-MB-231) [26]. Breast tumor-forming cells were decreased by inhibition of MAO-A activity with selective inhibitors [27]. The challenges linked to breast cancer treatment have encompassed various aspects, including the adverse effects of chemotherapy, treatment resistance, and the substantial expenses tied to radio and chemotherapy. Consequently, there has been a pressing need to explore novel therapeutic strategies for addressing breast cancer.

The current study is aimed at evaluating the efficacy of novel MAO-A inhibitors against different molecular types of breast cancer followed by molecular characterization of their possible underlying mechanism. To this end, a triple-negative breast cancer subtype, represented by MDA-231 cells, and a luminal subtype with ER/PR expression, represented by T47D cells, were chosen to profile their MAO-A expression pattern and probably the effect of its inhibition at the molecular level. The results of this research would contribute to the development of a new strategy in the management of breast cancer.

## 2. Materials and Methods

### 2.1. Cell Culture

Breast cancer cell lines, T-47D and MDA-MB-231 cells, were purchased from American Type Culture Collection (ATCC, USA). Cells were cultured in DMEM high glucose culture medium supplemented with 10% fetal bovine serum (FBS), 2 mM L-glutamine, 100 U/mL penicillin, and 0.1 mg/mL streptomycin. Cells were maintained in a humidified controlled temperature incubator set at 37°C, 95% humidity, and 5% CO_2_.

### 2.2. MAO-AIs

Based on the ligand-based drug design approach and structure-based drug design approach, certain HITs (J14, J15, J16, J17, J18, J19 (clorgyline), and J26 (moclobemide)) were identified by Bardaweel et al. as potential inhibitors for MAO-A, which were purchased from Sigma-Aldrich, Darmstadt, Germany. Also, novel MAO-A inhibitors (J20, J23, J24, and J25) were recently synthesized based on a pharmacophore modeling study [19]. All of the above-mentioned compounds were investigated for potential activity against lung cancer [19].

### 2.3. Cell Viability Assay

To assess the effect of MAO-AIs on the survival and proliferation of breast cancer cells, MTT colorimetric analysis was performed as previously described [28]. MDA-MB-231 and T47D cells were seeded into a 96-well plate, at a different seeding density per well depending on proliferation ability, doubling time, and target time of treatment. Then, cells were maintained at 37°C in a humidified 5% CO_2_ atmosphere. Cell viability was calculated as follows: cell viability (%) = [(optical density of viable cells in the test group)/optical density of viable cells in the negative control group] × 100%. Experiments were run at least 2-3 times independently. The concentration of drug required for 50% growth inhibition (IC_50_) was calculated using GraphPad Prism 9 software (GraphPad Software, San Diego, USA). To investigate the combined effect of MAO-A inhibitors (J14, J16, J19, and J25) with the anticancer (doxorubicin and raloxifene) used in breast cancer treatment, cells were treated with various concentrations of either J14, J16, J19, or J25 alone, combined with anticancer drugs (doxorubicin or raloxifene). The ratio of IC_50_ for each drug alone guided the selection of an appropriate combination ratio. As described earlier, the MTT test was used to determine cell viability after treatment time elapsed (48 hours). CompuSyn software (ComboSyn Inc., Paramus, NJ, USA) was used to determine the combination index (CI), which is based on Chou-Talalay's combination index theorem [29], and its formula is the sum of the ratio of the dose of each drug in the compound to the dose when used alone when the combination and compound produce 50% efficacy. CI values < 1, =1, and > 1 indicate synergism, additive, and antagonistic effects, respectively, as shown in Equation (1).

Equation ((1)) is Chou-Talalay's combination index theorem, where(*Dx*)1is the dose of drug 1 to produce 50% cell kill alone,(*D*)1is the dose of drug 1 to produce 50% cell kill in combination with(*D*)2,(*Dx*)2is the dose of drug 2 to produce 50% cell kill alone, and(*D*)2is the dose of drug 2 to produce 50% cell kill in combination with(*D*)1.

(1) $$\begin{array}{c} \text{CI}=\left(\frac{\left(D\right)1}{Dx}\right)+\left(\frac{\left(D\right)2}{Dx}\right). \end{array}$$

### 2.4. Wound Healing Assay

To assess the effect of MAO-AIs (J14, J16, J19, and J25) on the migration of MDA-MB-231 breast cancer cells, a wound healing assay was performed as previously described [30]. MDA-MB-231 were seeded in inserts (Ibidi, Germany) on a 24-well plate at a concentration of 45000 cells per insert side in 75 *μ*L media and incubated to reach 80-90% confluency almost for 24 hours. Afterward, inserts were removed, media were discarded, cells were washed with PBS and were incubated for 1-2 hours with 10 *μ*g/mL of mitomycin C to stop cell proliferation, the media were discarded, and cells were washed with fresh media twice prior treatment with IC_50_ and 0.5 IC_50_ concentrations of J14, J16, J19, and J25. Images were captured at zero, and after wound closure in untreated cells using the EVOS XL Core imaging system at 10x magnification. Digital images were analyzed for wound area and wound width (were calculated as the average distance between the edges of the wound) using ImageJ software ver. 1.53e. Percentage wound closure was calculated using Equation (2), and wound migration rate was calculated using Equation (3).

(2) $$\begin{array}{c} \%\text{wound closure}=\frac{A\;\left(t=0\right)-A\left(t=24\right)}{A\;\left(t=0\right)}\times 100\%. \end{array}$$

Equation ((2)) is the percentage of wound closure, where*A* (*t* = 0)is the wound area at zero time and*A* (*t* = 24)is the area width after 24 hours.

(3) $$\begin{array}{c} \text{Rate of migration }\left(\text{nm}/\text{hr}\right)=\frac{\text{wound intial width }\left(\text{zero time}\right)-\left(\text{wound final width }\left(24\text{ h}\right)\right.}{\text{duration of migration}}. \end{array}$$

Equation ((3)) is the rate of wound migration.

### 2.5. Cell Invasion Assay

This assay was done using Cell Biolabs, CytoSelect™ 96-Well Cell Invasion Assay (Basement Membrane, Fluorometric Format), CBA-112. MDA-MB-231 were seeded in a flask until 80-90% confluency. The invasion plate was allowed to warm up for 8-10 minutes at room temperature. The basement membrane layer of the membrane inserts was rehydrated with 100 *μ*L of warm, serum-free media. Next, it was incubated at room temperature for 1 hour. During the rehydration time, two cell suspensions containing 5 × 10^5^ cells/mL were prepared in a serum-free medium. IC_50_ and 0.5 IC_50_ of MAO-A inhibitors (J14, J16, J19, and J25) were added directly to the cell suspension. After removing the rehydration medium, the feeder tray wells were filled with 150 *μ*L of media containing 10% fetal bovine serum (as chemoattractant). Then, 100 *μ*L/well of cell suspension was added to the membrane chamber. After 48 hours of incubation, a 96-well cell harvesting tray was filled with 150 *μ*L of cell detachment solution per well. After cells/media from the top side of the membrane, the chamber was removed by inverting, and the membrane chamber was placed into the cell harvesting tray and incubated at 37°C for 30 minutes. The membrane chamber was removed after cells were collected by gently tilting the membrane chamber several times in the cell detachment solution. A sufficient 4× lysis buffer/CyQUANT GR dye solution was prepared by diluting the dye in 4× lysis buffer at a 1 : 75 ratio. 50 *μ*L of the dye solution was added to each well and incubated at room temperature for 20 minutes. Lastly, 150 *μ*L of the mixture was transferred to a 96-well black plate and was read with a fluorescent plate reader at 480 nm/520 nm [31].

### 2.6. Soft Agar Colony Formation Assay

To assess the effect of MAO-AIs (J14, J16, J19, and J25) on the anchorage-independent growth of MDA-MB-231 and T-47D breast cancer cells, soft agar colony formation assay was performed as previously described [30]. A base layer of 0.5% (*w*/*v*) noble agar was prepared in a 6-well plate by adding autoclaved at 120°C 1% agar solution to sterile filtered and warm 2× full DMEM (prepared immediately before use) in a 1 : 1 ratio and allowed to settle and solidify at room temperature. To properly cover the 6-well surface, each soft agar layer required 2 mL (1 mL media + 1 mL agar). For the upper 0.3% noble agar layer, we counted 1 × 10^4^ MDA-MB-231 and T47D cells, which all were pretreated for 48 h with either 0.5 IC_50_ or IC_50_ concentrations of J14, J16, J19, and J25. After that, the treated cells were mixed in a 1 : 1 ratio with 0.6% noble agar and poured on top of the base layer, which was then allowed to settle and solidify for 30 minutes at room temperature. Plates were incubated for 14 days in a CO_2_ incubator set at 37°C, 5% CO_2_, and 95% humidity and fortified gently with 300 *μ*L of DMEM full media twice weekly to prevent dissection of agar. Images were captured after 14-21 days at 4x and 20x magnifications (4x, 20x) using the EVOS XL Core imaging system (Invitrogen, USA). Colony size and colony numbers were measured using ImageJ software ver 1.53e.

### 2.7. Flow Cytometry

To assess the ability of MAO-AIs (J14, J16, J19, and J25) in induction apoptosis of T-47D and MDA-MB-231 breast cancer cells, annexin V-FITC/propidium iodide apoptosis assay was performed as previously described [32]. T-47D and MDA-MB-231 cells were seeded at a density of 4 × 10^5^ cells per well in 6-well plates, with a final volume of 5 mL of DMEM. The cells were then allowed to attach overnight in a humidified controlled temperature incubator set at 37°C, 95% humidity, and 5% CO_2_. Afterward, cells were treated with double IC_50_ concentration of J14, J16, J19, J25, and doxorubicin (positive control), and wells containing only fresh full DMEM were used as a negative control. After incubation time (48 hours) with treatment had elapsed, in a 5 mL flow tube, both floating and adhering cells (harvested using 500 *μ*L of trypsin) were collected and centrifuged for 10 minutes at 1400 rpm, 4°C, according to manufacturer protocol. The supernatant was discarded, and the cell pellet was resuspended in 500 *μ*L cold PBS and centrifuged to remove any remaining medium. Then, the pellets were resuspended again in 200 *μ*L of 1× binding buffer per tube. The cells were then stained with 5 *μ*L annexin V-FITC and incubated at room temperature for 5 minutes, followed by the addition of 10 *μ*L of propidium iodide (50 *μ*g/mL) to each tube. The samples were analyzed immediately using BD FACSCanto II flow cytometer (BD Biosciences, USA), and the analysis of the result was performed using BD FACSDiva software.

### 2.8. MAO-A Activity Assay

To assess the effect of MAO-AIs (J14, J16, J19, and J25) on the MAO-A activity of breast cancer cells, Monoamine Oxidase (MAO) Assay Kit (Abcam, UK) was performed as previously described [19]. T47D and MDA-MB-231 cells were seeded in a 6-well plate for 24 hours and then treated with IC_50_ of MAO-A inhibitors (J14, J16, J19, and J25) for 48 hours. After the treatment time had elapsed, a series of H_2_O_2_ standards 0, 200, 400, 600, 800, and 1000 pmol/well was prepared by adding 0, 2, 4, 6, 8, and 10 *μ*L of 0.1 mM H_2_O_2_, and the volume was adjusted up to 50 *μ*L/well with MAO assay buffer. Then, cells were detached using trypsin, centrifuged, and counted. For each sample, one million cells were used and were homogenized using 100 *μ*L of 0.1 mg/*μ*L MAO assay buffer. The homogenates were centrifuged at 4°C and 1400 rpm for 10 minutes, and the supernatants were collected. Next, 10 *μ*L of MAO-B inhibitor (selegiline) was added to 1-40 *μ*L of supernatant, and the volume was adjusted to 50 *μ*L/well with MAO assay buffer. Just before use, positive control was prepared by adding 1-4 *μ*L of positive control solution into desired wells and completing the volume up to 50 *μ*L/well with MAO assay buffer. Then, the plate was incubated at 25°C for 10 minutes. 50 *μ*L of reaction mix (MAO assay buffer, developer, MAO substrate (tyramine), and probe) was added into each standard, sample, and positive control well. 50 *μ*L of background reaction mix (MAO assay buffer, developer, and probe) per well was added into the background control sample wells. Finally, fluorescence readings were taken after 60 minutes of incubation at room temperature, at Ex/Em = 535/587 nm.

### 2.9. Real-Time Polymerase Chain Reaction (PCR)

To assess the effect of MAO-AIs (J14 and J16) on the *MAO-A*, *Bcl-2*, *VEGF*, and GAPDH gene expressions of MDA-MB-231 and T-47D breast cancer cells, RNA extraction using Direct-zol™ RNA Miniprep Plus Kit, complementary DNA synthesis, and the Applied Biosystems 7900 real-time PCR detection systems (Applied Biosystems, USA) was used to perform quantitative real-time PCR using SYBR Green Real-Time PCR Master Mix as previously described [33]. Primer sequence and their optimized annealing temperature (Ta) are shown in Table 1. Using a 20 *μ*L sample (cDNA) volume per reaction, recommended thermal cycling included one initial denaturation cycle for 15 minutes at 95°C, followed by 45 cycles of 15 seconds at 95°C, 30 seconds at Ta°C, and 30 seconds at 72°C. The MAO elongation step was carried out for 55 seconds at 72°C. Changes of expression were normalized against the GAPDH housekeeping gene using *ΔΔCt* method.

### 2.10. Statistical Analysis

Data analysis was performed using GraphPad Prism software (GraphPad Prism version 9.0.0 for Windows, GraphPad Software, San Diego, California, USA). The differences between treatment groups were determined by independent sample *t*-test, one-way ANOVA, or two-way ANOVA. Data are expressed as mean ± SD, and *p* < 0.05 was considered a statistically significant difference.

## 3. Results and Discussion

Cancer is the second main cause of death in the United States and is considered a major public health problem worldwide [34]. Breast cancer is the leading cause of death among women aged 20 to 49 years [34]. In 2020, 2,261,419 women in the world were diagnosed with breast cancer and 684,996 died in the same year [35]. The MAO-AIs have effective antiproliferative activity against gliomas [16], prostate [13, 36], colorectal cancer [18], and lung cancer cells [19]. MAO-AI (clorgyline, J19) reduced the expression of MAO-A gene in prostate cancer [13].

In this study, MTT assay was performed after exposing MDA-MB-231 and T-47d cell lines to increasing concentrations of MAO-A inhibitors for either 24, 48, or 72 h. Compounds J14, J16, J19, and J25 have shown considerable antiproliferative activities, and the treated cells had reduced cell viability compared to the untreated control cells (Table 1 Supplementary). Specifically, J14 exhibited potent antiproliferative activities against MDA-MB-231 and T-47d with IC_50_ values of 12.39 *μ*M and 7.6 *μ*M, respectively. In addition, J16 demonstrated antiproliferative activities against MDA-MB-231 and T-47d with IC_50_ values of 30.6 *μ*M and 28.52 *μ*M, respectively. On the other hand, J19 had significant antiproliferative activities against MDA-MB-231 and T-47d with IC_50_ values of 162.8 *μ*M and 157.8 *μ*M, respectively. Our results come in good agreement with Satram-Maharaj et al.'s findings which reported that clorgyline (J19) significantly inhibited MAO-A catalytic activity in MCF7 and MDA-MB-231 cells [26]. Among the novel synthesized compounds, J25 displayed noticeable antiproliferative activities against MDA-MB-231 and T-47d with IC_50_ values of 184.6 *μ*M and 148.7 *μ*M, respectively. The similarity of antiproliferative effects of MAO-AIs in MDA-MB-231 (ER-negative), and T-47D (ER-positive) cells may suggest that the role of MAO-A in breast cancer progression is independent of estrogen receptor (ER) expression status [26]. Interestingly, J15, J17, J18, J20, J24, and J26 did not show any antiproliferative activity on treated cells within the examined concentration range (Table 3 Supplementary). Noteworthy, it appears that the ability of the MAO-A inhibitors to affect the proliferation of breast cancer cells is not only mediated through their MAO-A inhibition but also crosslinks with several signaling pathways in the cancer cell, suggesting new roles of MAO-A at the molecular level.

Doxorubicin is associated with dose-dependent cardiac cytotoxicity, which limits its clinical usefulness [37]. Also, breast cancer resistance to doxorubicin is commonly associated with reduced intracellular drug concentrations via the increase in the activity of P-glycoprotein efflux pumps [38]. Studies have reported that MAO-AI treatment significantly downregulated P-glycoprotein expression [39]. The combination of J14, J16, J19, and J25 with chemotherapy (doxorubicin or raloxifene) reduced the effective dose of the anticancer agents needed to yield an antiproliferative effect in breast cancer cells (synergistic effect, CI < 1), which may reduce the dose resistance and the dose-dependent toxicity (Table 2 Supplementary).

**Figure 1:**
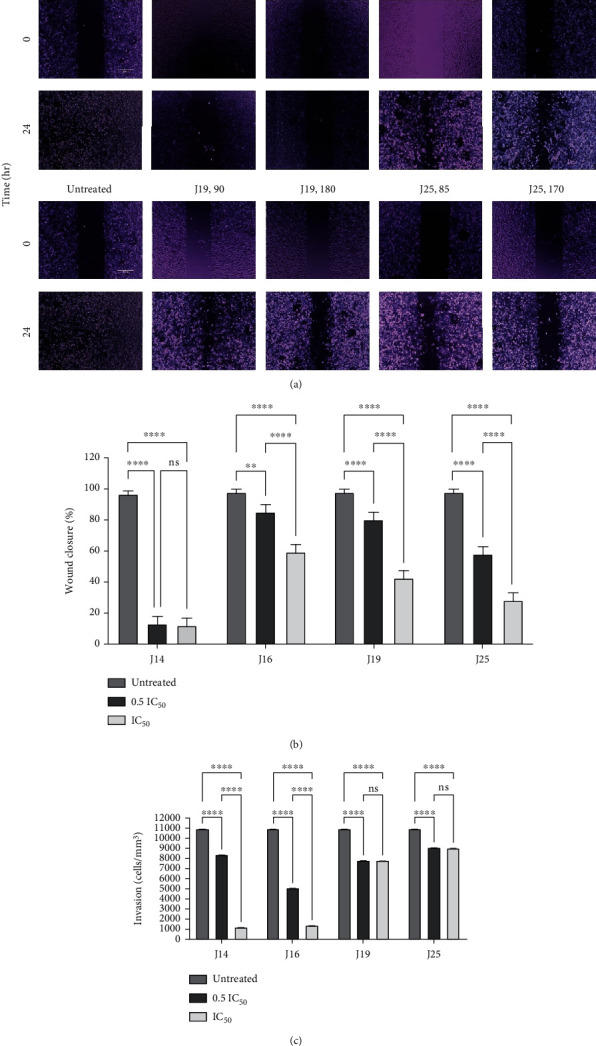
The effect of MAO-AIs (J14, J16, J19, and J25) treatment on MDA-MB-231 cell (a) migration and (b) its analysis and (c) invasion. Experiments were run in duplicate for at least two independent trials (*n* = 4). The standard deviation of all IC_50_ values did not exceed 5%. IC50: the 50% inhibitory concentration; *p* value < 0.05 expresses significantly different from respective untreated condition; ^ns^*p* > 0.05 (not significant); ^∗^*p* ≤ 0.05; ^∗∗^*p* ≤ 0.01; ^∗∗∗^*p* ≤ 0.001; ^∗∗∗∗^*p* ≤ 0.0001 (according to GraphPad Prism 9).

Moreover, MDA-MB-231 cells were used in the migration and invasion assays, due to their high migratory rate [40]. The IC_50_ and sub-IC_50_ concentrations of J14, J16, J19, and J25 significantly (*p* value < 0.0001) inhibited the migration (Figure 1(b)) and invasion (Figure 1(c)) of MDA-MB-231 cells compared to untreated cells. Wound images are shown in (Figure 1(a)). In contrast, MAO-AI (clorgyline) significantly increased invasiveness through Matrigel and the migratory capacity of MDA-MB-231 [26].

**Figure 2:**
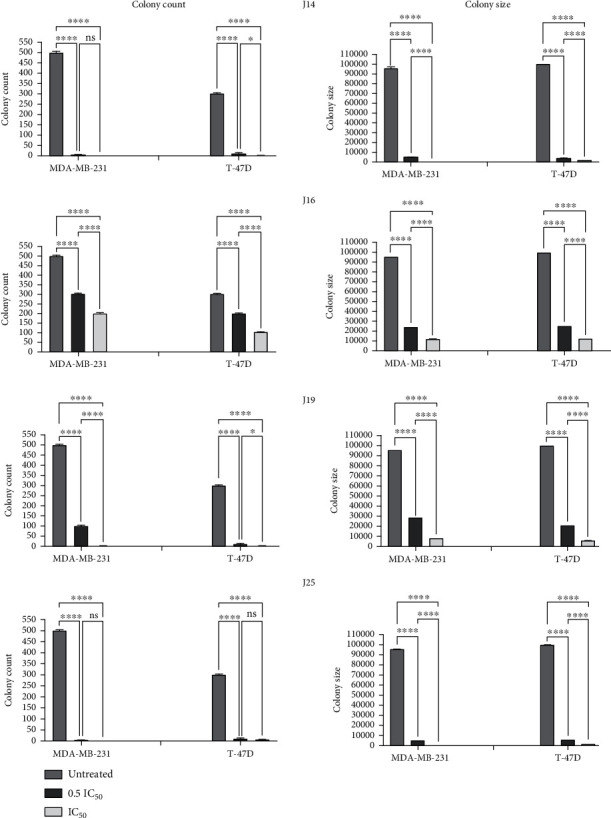
Effect of MAO-AIs (J14, J16, J19, and J25) treatment on colony count and colony size of MDA-MB-231 and T-47D breast cancer cells. Colony size was measured using particle analysis upon identifying the colony color threshold through ImageJ software (ver. 1.53e.). *p* value < 0.05 indicates statistical significance in comparison to untreated control; ^ns^*p* > 0.05 (not significant); ^∗^*p* ≤ 0.05; ^∗∗^*p* ≤ 0.01; ^∗∗∗^*p* ≤ 0.001; ^∗∗∗∗^*p* ≤ 0.0001 (according to GraphPad Prism 9).

Interestingly, the macrophage colony-stimulating factor (MCSF) is found to be overexpressed in breast cancer [41]. MCSF overexpression and its receptor are usually associated with tumor-poor prognosis [41]. J14, J16, J19, and J25 treatments for 48 h significantly (*p* value < 0.0001) inhibited the ability of MDA-MB-231 and T-47D breast cancer cells to form colonies by reducing the number and size of colonies compared to untreated control cells (Figure 2). Images for colonies were taken at different magnifications (4x and 20x) on day 14 (Figure 1 supplementary). These findings are compatible with the previously reported literature where MDA-MB-231 cells' ability to form colonies was inhibited when treated with MAO-AI (clorgyline, J19) [26].

**Figure 3:**
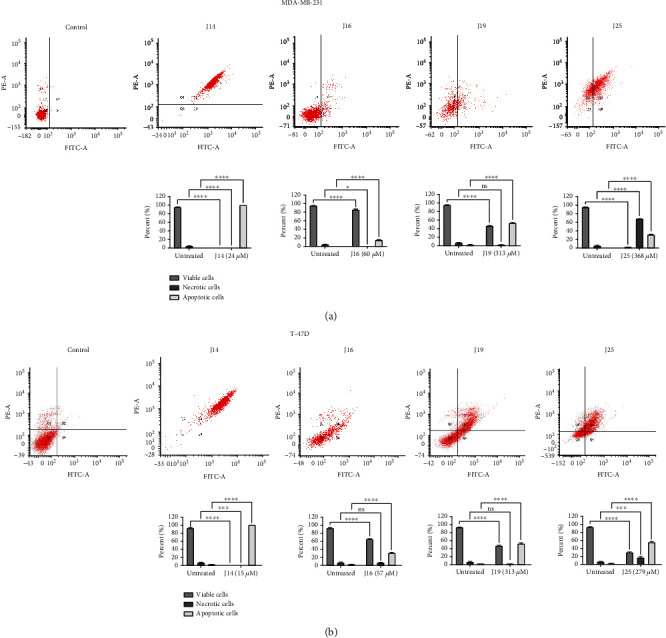
Dot plot for annexin V-FITC/PI staining expressing the apoptotic effect of double IC_50_ of MAO-AIs (J14, J16, J19, and J25) treatment for 48-hour treatment against (a) MDA-MB-231 and (b) T-47D cells and its quantitative comparison of each cell phase, where Q3 showed viable cells, Q1 necrotic cells, Q2 late apoptotic, and Q4 early apoptotic. Percentages of healthy, apoptotic, and necrotic cells expressed as mean, SD did not exceed 5%. *P*-value <0.05 indicates statistical significance in comparison to untreated control, while asterisk: ^ns^*p* > 0.05 (not significant); ^∗^*p* ≤ 0.05; ^∗∗^*p* ≤ 0.01; ^∗∗∗^*p* ≤ 0.001; ^∗∗∗∗^*p* ≤ 0.0001 (according to GraphPad Prism 9).

The common mechanism of antiproliferative agents is the induction of apoptosis (programmed cell death) [42]. Treatment of MDA-MB-231 and T-47D cells with double IC_50_ concentrations of J14, J16, J19, and J25 resulted in a significant (*p* value < 0.0001) increase in early and late apoptosis (*Q*2 + *Q*4 regions presented in the dot plot) in breast cancers exposed compared to untreated control groups. On the other hand, necrosis was induced in MDA-MB-231 cells upon treatment with J19 and J25 and in T-47D cells upon treatment with J25, as shown in Figure 3(a) for MDA-MB-231 cells and Figure 3(b) for T-47D cells.

**Figure 4:**
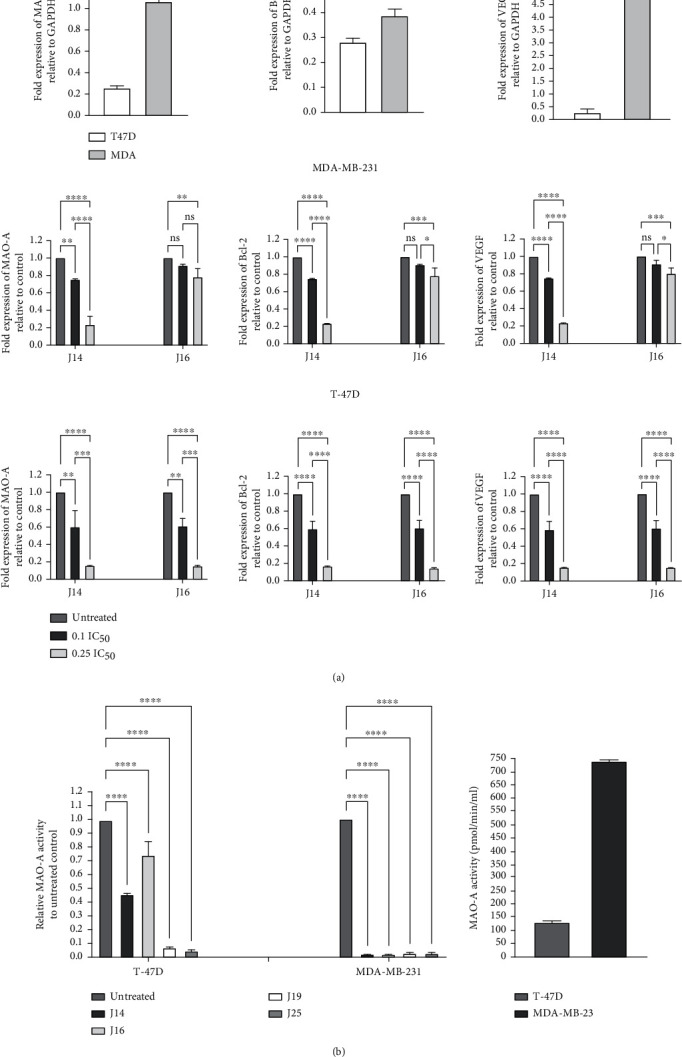
The effect of MAO-AIs on (a) *MAO-A*, *BCL-2*, and *VEGF* genes and (b) MAO-A activity expression in MDA-MB-231 and T-47D breast cancer cell lines. Fold difference was expressed as mean ± SD and was measured using ΔΔCt method; *p* value < 0.05 expresses significantly different from respective untreated cells status; ^ns^*p* > 0.05 (not significant); ^∗^*p* ≤ 0.05; ^∗∗^*p* ≤ 0.01; ^∗∗∗^*p* ≤ 0.001; ^∗∗∗∗^*p* ≤ 0.0001 (according to GraphPad Prism 9). *MAO-A*: monoamine oxidase A; *Bcl-2*: the gene for B-cell lymphoma 2; *VEGF*: the gene for vascular endothelial growth factor; GAPDH: the gene for glyceraldehyde 3-phosphate dehydrogenase.

Vascular endothelial growth factor (*VEGF*) plays an important role in tumor angiogenesis, growth, and metastasis [43]. It is overexpressed in breast cancer [44]. One of the therapy targets in breast cancer is antiangiogenesis [45]. J14 and J16 significantly inhibited *VGEF* gene expression. It was consistent with its observed effect on tumor cells as growth and metastasis suppressors. *Bcl-2* gene (apoptotic regulator) inhibits apoptosis [46]. J14 and J16 significantly decreased *Bcl-2* gene expression, which is consistent with the apoptotic effect of these MAO-AIs. Breast cancer cells that developed anticancer drug resistance appeared to have elevated MAO-A expression [27]. Our results suggest that *MAO-A*, *Bcl-2*, and *VEGF* are being overexpressed in MDA-MB-231 cells compared to T-47D cells. Compared to the control groups (untreated cells), treatment with J14 and J16 resulted in a significant (*p* value < 0.01) reduction of the expression of *MAO-A*, *Bcl-2*, and *VEGF* in MDA-MB-231 and T-47D breast cancer cells (Figure 4(a)).

Finally, when tested for the catalytic activity, MAO-A enzyme activity in MDA-MB-231 cells appears to be higher than in T-47D cells. Treatment of MDA-MB-231 and T-47D breast cancer cells with IC_50_ of J14, J16, J19, and J25 for 48 hours resulted in significant (*p* value < 0.0001) inhibition of MAO-A activity in comparison to untreated control (Figure 4(b)).

## 4. Conclusion

The present study demonstrated the potential usefulness of MAO-A inhibitors as antiproliferative, antimigratory, and synergistic anticancer agents in the treatment of breast cancer. Moreover, the current study provided the first evidence of MAO-A involvement in the regulation of several genes in human breast cancer; modulation of *MAO-A*, *VEGF*, and *Bcl-2* genes may indicate the multisignaling pathways in which MAO-A is involved in the cancer cellular compartment.

## Figures and Tables

**Table 1:** Primers' forward and reverse sequences with their optimized annealing temperature.

Primer	Primer sequence	Ta (°C)
*MAO-A*	Forward: 5-GCCAAGATTCACTTCAGACCAGAG-3	59
	Reverse: 5-TGCTCCTCACACCAGTTCTTCTC-3	

*Bcl-2*	Forward: 5-TTGTGGCCTTCTTTGAGTTCGGTG-3	59
	Reverse: 5-GGTGCCGGTTCAGGTACTCAGTCA-3	

*VEGF*	Forward: 5-CTACCTCCACCATGCCAAGT-3	*59*
	Reverse: 5-GCAGTAGCTGCGCTGATAGA-3	

*GAPDH*	Forward: 5-ACAACTTTGGTATCGTGGAAGG-3	58
	Reverse: 5-GCCATCACGCCACAGTTTC-3	

Ta: annealing temperature; MAO-A: the gene for monoamine oxidase A enzyme; Bcl-2: the gene for B-cell lymphoma 2; VEGF: the gene for vascular endothelial growth factor; GAPDH: the gene for glyceraldehyde 3-phosphate dehydrogenase.

## Data Availability

The data that support the findings of this study are available from the corresponding author, Sanaa K Bardaweel, upon reasonable request.

## Supplementary Materials

Supplementary 1 Figure 1 supplementary describes the images for colonies that were taken at different magnifications (4x and 20x) on day 14.

Supplementary 2 Table 1 supplementary describes the IC_50_ values for MAO-AIs against MDA-MB-231 and T47D breast cancer cells. MTT assay was carried out for 24-, 48-, and 72-hour treatment duration.

Supplementary 3 Table 2 supplementary describes the effect of the combination of MAO-AIs with doxorubicin or raloxifene on IC_50_ values in treatment of MDA-MB-231 and T-47D breast cancer cells for 48-hour duration.

Supplementary 4 Table 3 supplementary describes the 50% inhibitory concentration (IC_50_) values for MAO-A inhibitors in MDA-MB-231 and T47D breast cancer cells at 24, 48, and 72 h.
