# Supplementary material for: Molecular Investigation of the Antitumor Effects of Monoamine Oxidase Inhibitors in Breast Cancer Cells
Source: Biomed Res Int. 2023 Oct 5;2023:2592691. doi: 10.1155/2023/2592691 (PMC10569896; doi:10.1155/2023/2592691)
Supplement: Supplementary 1 — Figure 1 supplementary describes the images for colonies that were taken at different magnifications (4x and 20x) on day 14. [file 2592691.f1.pdf]

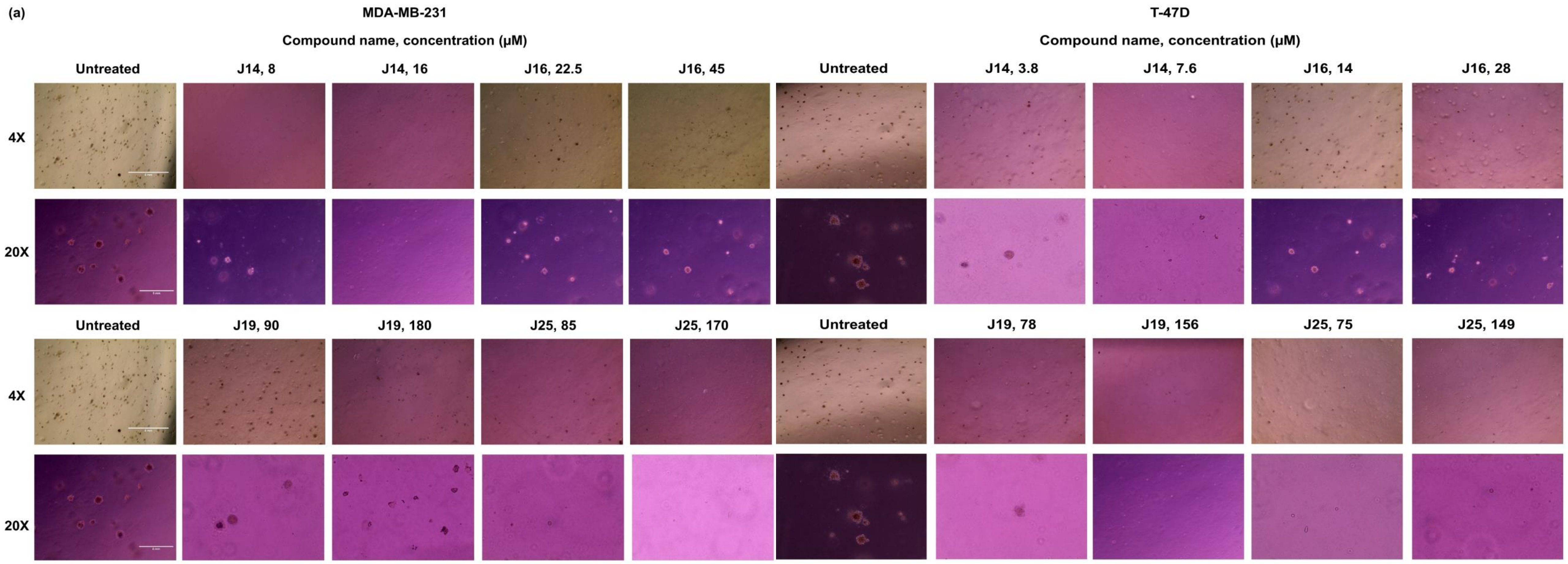
(a)
MDA-MB-231
Compound name, concentration (μM)
Untreated
J14, 8
J14, 16
J16, 22.5
J16, 45
4X
20X
Untreated
J19, 90
J19, 180
J25, 85
J25, 170
4X
20X
T-47D
Compound name, concentration (μM)
Untreated
J14, 3.8
J14, 7.6
J16, 14
J16, 28
Untreated
J19, 78
J19, 156
J25, 75
J25, 149
