# Supplementary material for: Molecular Investigation of the Antitumor Effects of Monoamine Oxidase Inhibitors in Breast Cancer Cells
Source: Biomed Res Int. 2023 Oct 5;2023:2592691. doi: 10.1155/2023/2592691 (PMC10569896; doi:10.1155/2023/2592691)
Supplement: Supplementary 2 — Table 1 supplementary describes the IC50 values for MAO-AIs against MDA-MB-231 and T47D breast cancer cells. MTT assay was carried out for 24-, 48-, and 72-hour treatment duration. [file 2592691.f2.pdf]

Table 1 Supplementary The IC_{50} values for MAO-AIs against MDA-MB-231 and T47D breast cancer cells.

Cell line	MDA-MB-231	T-47D	Cell line	MDA-MB-231	T-47D
	J14, IC_{50} (µM, mean ± SD)			J19, IC_{50} (µM, mean ± SD)	
24 h	(16.07±2.9)	(10.27±1.62)	24 h	(180±4.4)	(179.1±4.6)
48 h	(12.39±2.6)	(7.6±2.1)	48 h	(162.8±5.1)	(156.8±4.9)
72 h	(10.44±3)	(5.6±1.1)	72 h	(169.5±4.7)	(170±5)
	J16, IC_{50} (µM, mean ± SD)			J25, IC_{50} (µM, mean ± SD)	
24 h	(45.57±4)	(45.6±4.62)	24 h	(169.4±5)	(164.8±4)
48 h	(30.6±5.1)	(28.52±5)	48 h	(184.6±5)	(148.7±5)
72 h	(22±4.2)	(18.3±4.9)	72 h	(115±3)	(170.7±3)

MTT assay was carried out for 24, 48, 72 hours treatment duration. Experiments were run in triplicates for at least three independent trials (n=9). standard deviation (SD) of all IC50 values did not exceed 5%. IC50: the 50% inhibitory concentration: h: hour; µM: micromolar.
