# Supplementary material for: Molecular Investigation of the Antitumor Effects of Monoamine Oxidase Inhibitors in Breast Cancer Cells
Source: Biomed Res Int. 2023 Oct 5;2023:2592691. doi: 10.1155/2023/2592691 (PMC10569896; doi:10.1155/2023/2592691)
Supplement: Supplementary 3 — Table 2 supplementary describes the effect of the combination of MAO-AIs with doxorubicin or raloxifene on IC50 values in treatment of MDA-MB-231 and T-47D breast cancer cells for 48-hour duration. [file 2592691.f3.pdf]

Table 2 Supplementary The effect of combination MAO-AIs with Doxorubicin or Raloxifene on IC_{50} values in treatment MDA-MB-231 and T-47D breast cancer cells for 48 hours duration.

MDA-MB-231									
Compound	Ratio	IC_{50} (µm)	CI	Fold of reduction	Compound	Ratio	IC_{50} (µm)	CI	Fold of reduction
Doxorubicin alone	NA	2	NA	NA	Raloxifene alone	NA	27	NA	NA
Doxorubicin:J14	1:5	1	0.92	2	Raloxifene:J14	5:1	18	0.97	1.5
Doxorubicin:J16	1:5	1.6	0.99	1.3	Raloxifene:J16	5:1	22	0.96	1.2
Doxorubicin:J19	1:100	0.81	0.91	2.5	Raloxifene:J19	1:10	8.1	0.8	3.3
Doxorubicin:J25	1:100	0.92	0.96	2.2	Raloxifene:J25	1:10	9.2	0.84	2.9
T-47D									
Doxorubicin alone	NA	2.2	NA	NA	Raloxifene alone	NA	28	NA	NA
Doxorubicin:J14	1:5	0.3	0.33	7.3	Raloxifene:J14	5:1	13	0.91	2.2
Doxorubicin:J16	1:5	0.3	0.19	7.3	Raloxifene:J16	5:1	14	0.71	2.0
Doxorubicin:J19	1:100	0.6	0.66	3.7	Raloxifene:J19	1:10	10	0.99	2.8
Doxorubicin:J25	1:100	0.4	0.45	5.5	Raloxifene :J25	1:10	9	0.9	3.1

Experiments were run in triplicates for at least three independent trials (n=9). standard deviation of all IC50 values did not exceed 5%. IC50: the 50% inhibitory concentration; NA: not applicable; h: hour; µM: micromolar.
