# Supplementary material for: Molecular Investigation of the Antitumor Effects of Monoamine Oxidase Inhibitors in Breast Cancer Cells
Source: Biomed Res Int. 2023 Oct 5;2023:2592691. doi: 10.1155/2023/2592691 (PMC10569896; doi:10.1155/2023/2592691)
Supplement: Supplementary 4 — Table 3 supplementary describes the 50% inhibitory concentration (IC50) values for MAO-A inhibitors in MDA-MB-231 and T47D breast cancer cells at 24, 48, and 72 h. [file 2592691.f4.pdf]

Table 3 Supplementary The 50% inhibitory concentration (IC_{50}) values for MAO-A inhibitors in MDA-MB-231 and T47D breast cancer cells.

Cell line	MDA-MB-231	T-47D	Cell line	MDA-MB-231	T-47D
	J15, IC_{50} (µM, mean ± SD)			J17, IC_{50} (µM, mean ± SD)	
24 h	>1000	>1000	24 h	>1000	>1000
48 h	>1000	>1000	48 h	>1000	>1000
72 h	>1000	>1000	72 h	>1000	>1000
	J18, IC_{50} (µM, mean ± SD)			J20, IC_{50} (µM, mean ± SD)	
24 h	>1000	>1000	24 h	>1000	>1000
48 h	>1000	>1000	48 h	>1000	>1000
72 h	>1000	>1000	72 h	>1000	>1000
	J23, IC_{50} (µM, mean ± SD)			J24, IC_{50} (µM, mean ± SD)	
24 h	>1000	>1000	24 h	>1000	>1000
48 h	>1000	>1000	48 h	>1000	>1000
72 h	>1000	>1000	72 h	>1000	>1000
	J26, IC_{50} (µM, mean ± SD)				
24 h	>1000	>1000			
48 h	>1000	>1000			
72 h	>1000	>1000			

MTT assay was carried out for 24, 48, 72 hours treatment duration. Experiments were run in triplicates for at least three independent trials (n=9). standard deviation (SD) of all IC50 values did not exceed 5%. IC50: the 50% inhibitory concentration: h: hour; µM: micromolar.
